# Supplementary material for: PIP2 promotes conformation-specific dimerization of the EphA2 membrane region
Source: J Biol Chem. 2020 Dec 10;296:100149. doi: 10.1074/jbc.RA120.016423 (PMC7900517; doi:10.1074/jbc.RA120.016423)
Supplement: Supplementary Figures [file mmc1.pdf]

Supporting Information

PIP2 promotes conformation-specific dimerization of the EphA2 membrane region

Katherine M. Stefanski, Charles M. Russell, Justin M. Westerfield, Rajan Lamichhane, Francisco N. Barrera

Supplementary Figures:

Supplemental Figure 1. CD of TMJM at 50:1 and OCD at 300:1

Supplemental Figure 2. TEM images and size distributions of SMALPs

Supplemental Figure 3. HPLC and MADLI-TOF of TMJM-Alexa488

Supplemental Figure 4. Controls for immobilization of SMALPs on slides via biotin-streptavidin linkage and SMALP composition.

Supplemental Figure 5. Percent larger oligomers of TMJM in SMALPs.

Supplemental Figure 6. Tryptophan fluorescence spectra and spectral maxima.

Supplemental Figure 7. Tryptophan-DNS FRET spectra and efficiencies at different acceptor concentrations.

Supplemental Figure 8. OCD of TMJM in 22:1 PC and 14:1 PC with PIP_2.

Supplemental Figure 9. Peptide recovery and calcium influx controls.

Supplemental Figure 10. Full SDS-PAGE gel of TMJM crosslinking.

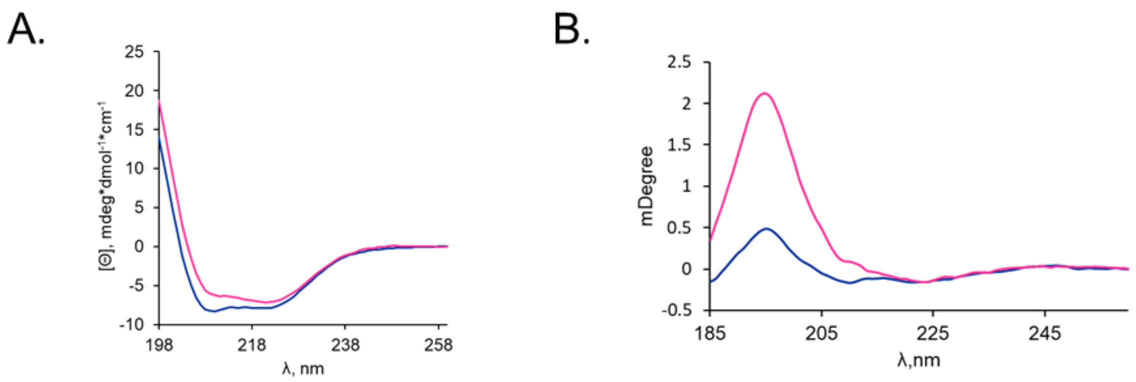

Supplemental Figure 1. **A.** CD of TMJM in 22:1 PC (fuchsia) and 14:1 PC (navy) liposomes at a lipid to peptide ratio of 50:1 showing that the secondary structure in both lipids is α-helical. **B.** OCD of TMJM in 14:1 PC (navy) and 22:1 PC (fuchsia) at a lipid to peptide ratio of 300:1.

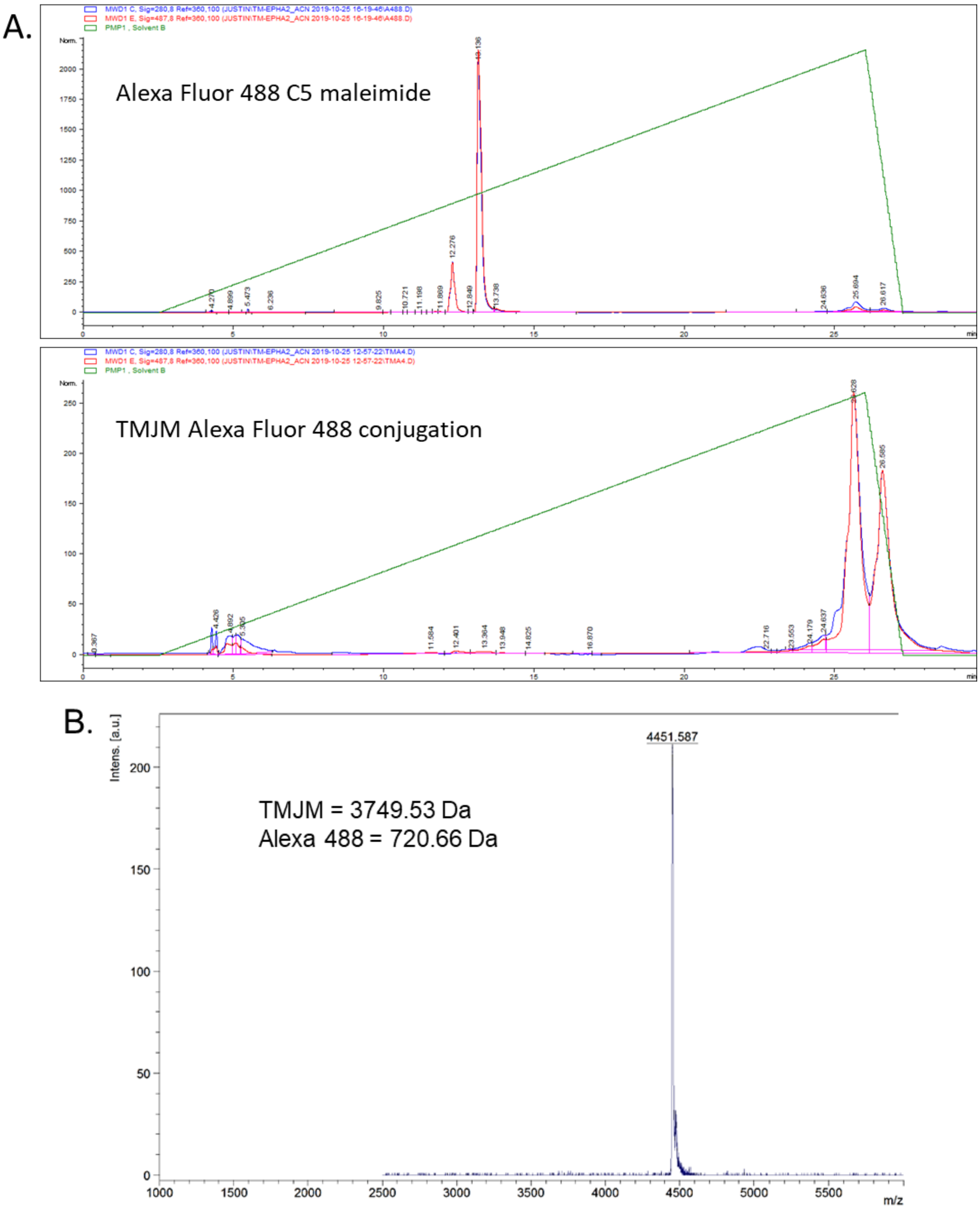

Supplemental Figure 2. A. HPLC and MADLI-TOF of TMJM-Alexa488 conjugation. A. HPLC chromatogram of purification showing free dye eluting at 10-15 minutes (top) and TMJM-Alexa Fluor 488 (bottom) eluting at 25+ min (25.4 – 27 min were collected). **B.** MALDI-TOF of TMJM-Alexa Fluor 488 showing a single, purified peak.

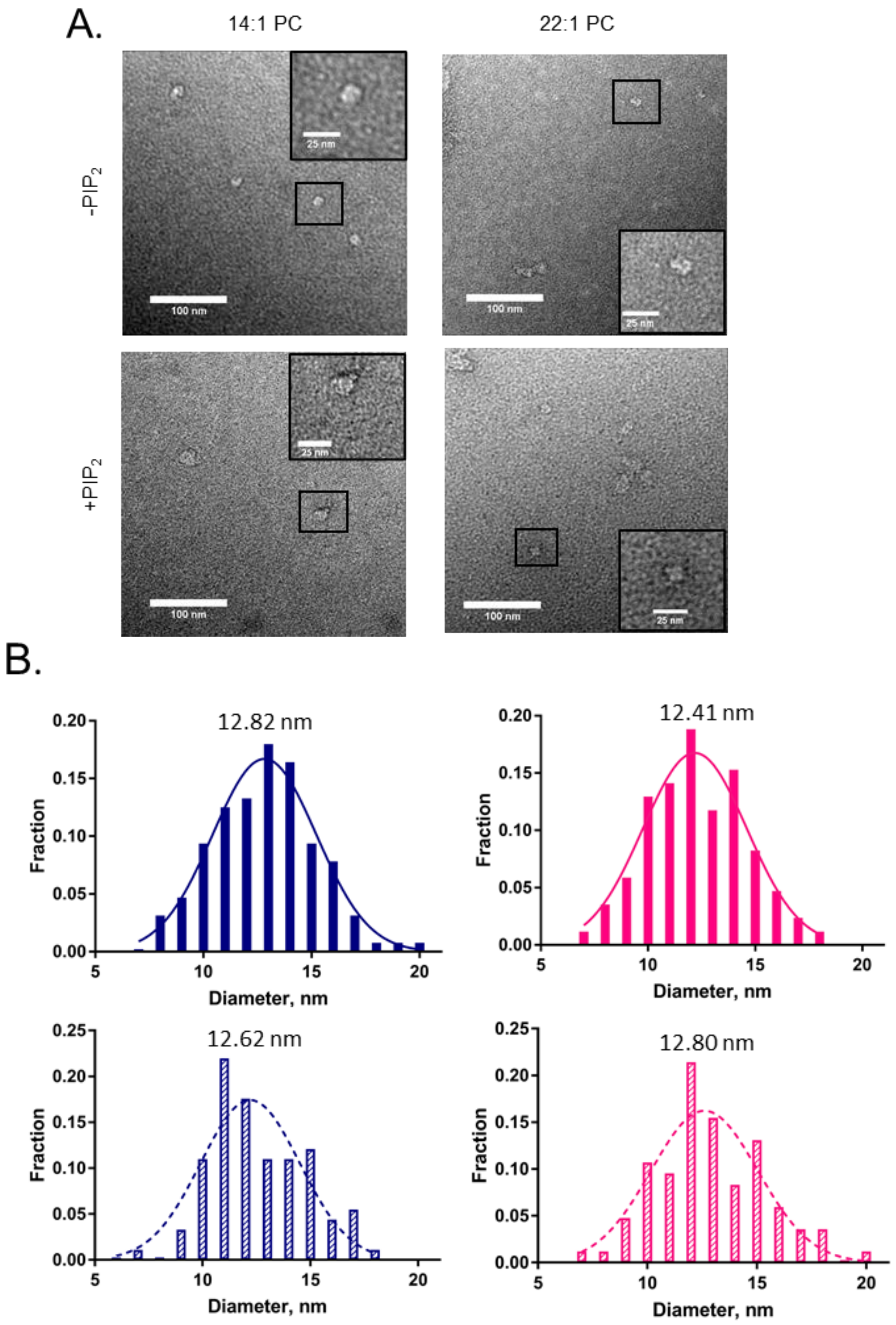

Supplemental Figure 3. A. Representative TEM images of SMALPs comprised of 22:1 and 14:1 PC ± 3% PIP_2. Scale bars in large images are 100 nm. Scale bars in insets are 25 nm. **B.** Histograms of SMALP diameters with Gaussian fits. Top Right: 22:1 PC (solid bars) and Bottom Right: 22:1 PC + PIP_2 (cross hatch). Top Left: 14:1 PC (solid bars) and Bottom Left: 14:1 PC + PIP_2 (cross hatch). Data are from 3-4 independent SMALP preparations for each lipid composition. 80-100 SMALPs were measured for each lipid composition. Value above each panel is midpoint of distribution.

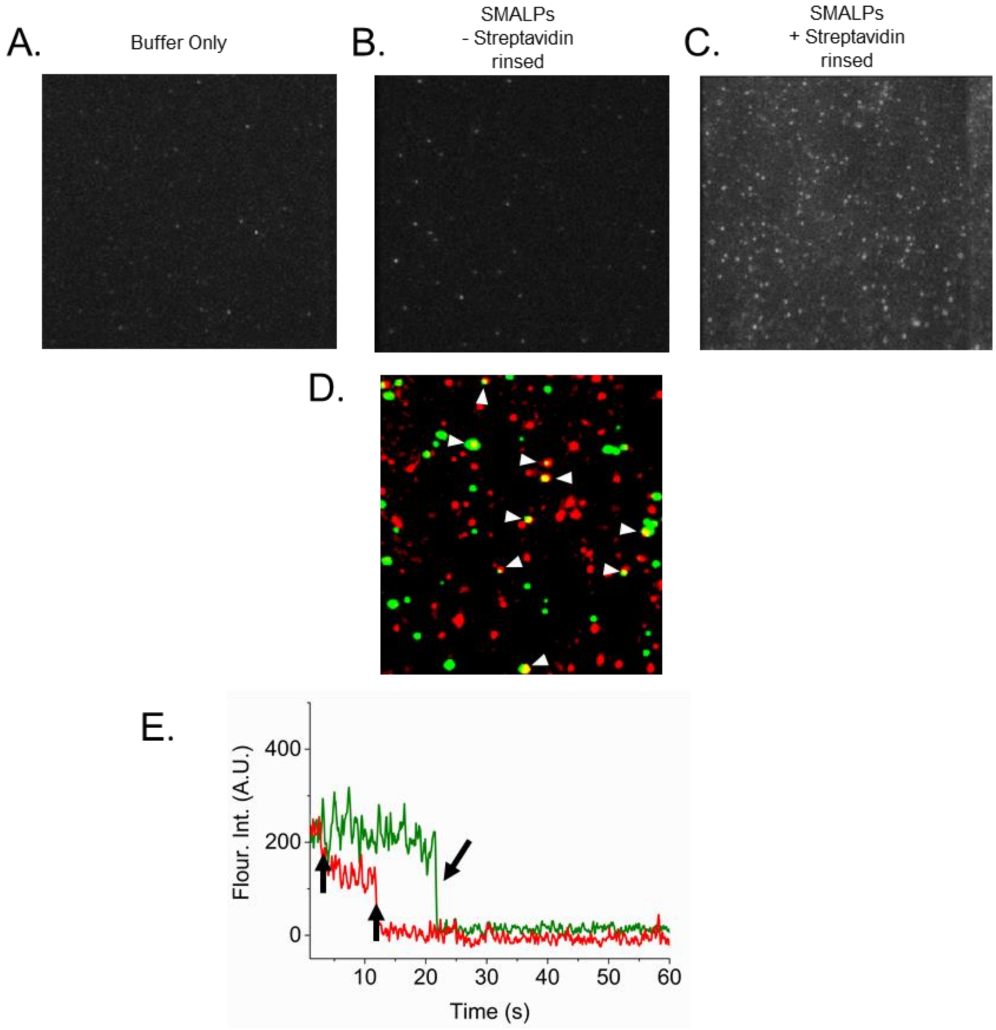

Supplemental Figure 4. Controls for immobilization of SMALPs on slides via biotin-streptavidin linkage and SMALP composition. A. Control biotinylated slide with buffer only. **B.** A biotinylated slide without streptavidin was incubated for 10 minutes with SMALPs containing 3% biotin-PE and TMJM Alexa Fluor 488 and rinsed. Images show no non-specific immobilized SMALPs. **C.** A biotinylated slide was incubated with 0.2 mg/mL streptavidin for 10 min followed by incubation with SMALPs containing biotin-PE and TMJM Alexa488 then rinsed. Image shows immobilized SMALPs. **D.** Co-localization of SMALPs with PIP_2 Bodipy FL (left) and TMJM Cy5 (right) simultaneously excited. White arrows highlight examples of SMALPs containing both PIP_2 and TMJM. **E.** Representative fluorescent trace from co-localization data in panel D showing TMJM Cy5 (red) with two photobleaching steps (black arrows) and fluorescence from PIP_2 Bodipy FL (green) showing one photobleaching step (black arrow).

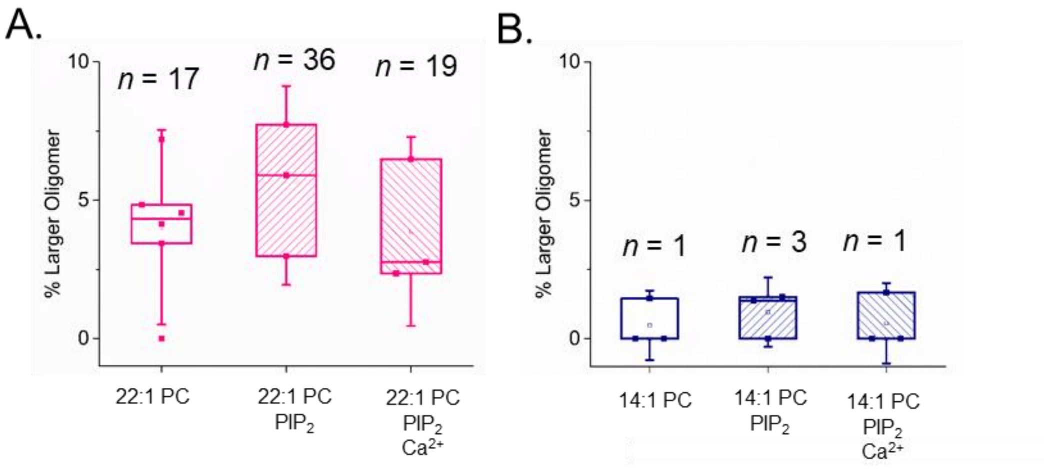

Supplemental Figure 5. A. and **B.** Percentage of peptide in larger oligomers in 22:1 PC and 14:1 PC SMALPs with and without 3% PIP_2 and 5 mM Ca^{2+} via SM-photobleaching experiments. Data are from 3-6 independent experiments. *n* = number of traces counted with 3 or more steps.

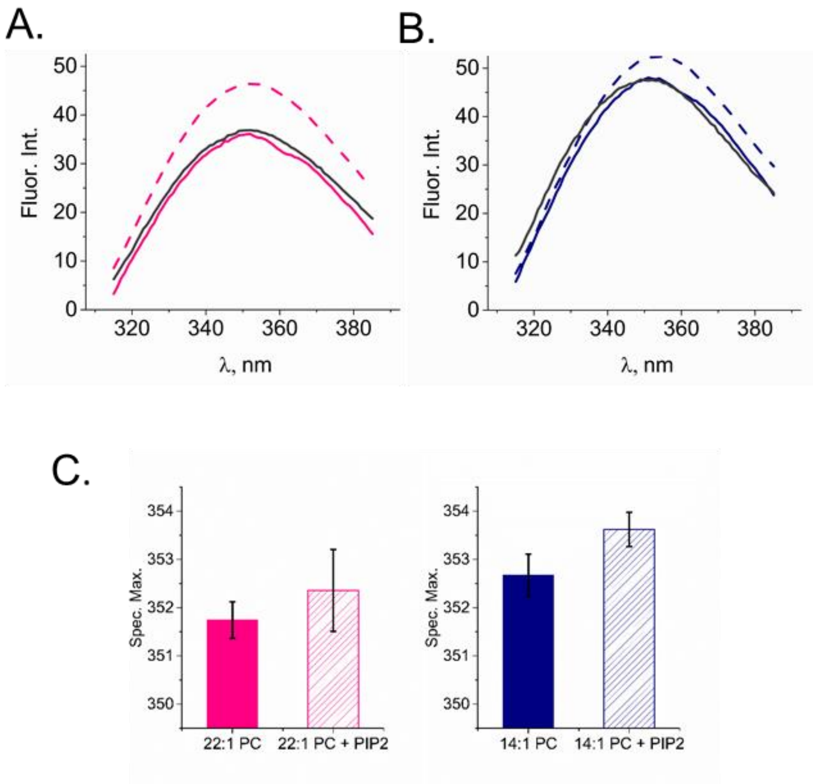

Supplementary Figure 6. A. Trp emission spectra in 22:1 PC liposomes (solid fuchsia line), with 3% PIP_2 (dashed line) and 3% PIP_2 with Ca^{2+} (gray line) (curves are averages of 3 independent experiments). **B.** Trp emission spectra in 14:1 PC liposomes (solid navy line), with 3% PIP_2 (dashed line) and 3% PIP_2 with Ca^{2+} (gray line). Curves are averages of 3 independent experiments. **C.** Tryptophan fluorescence spectral max in 14:1 PC and 22:1 PC liposomes with and without 3% PIP_2. Bars are means ± S.D. from 3 independent experiments.

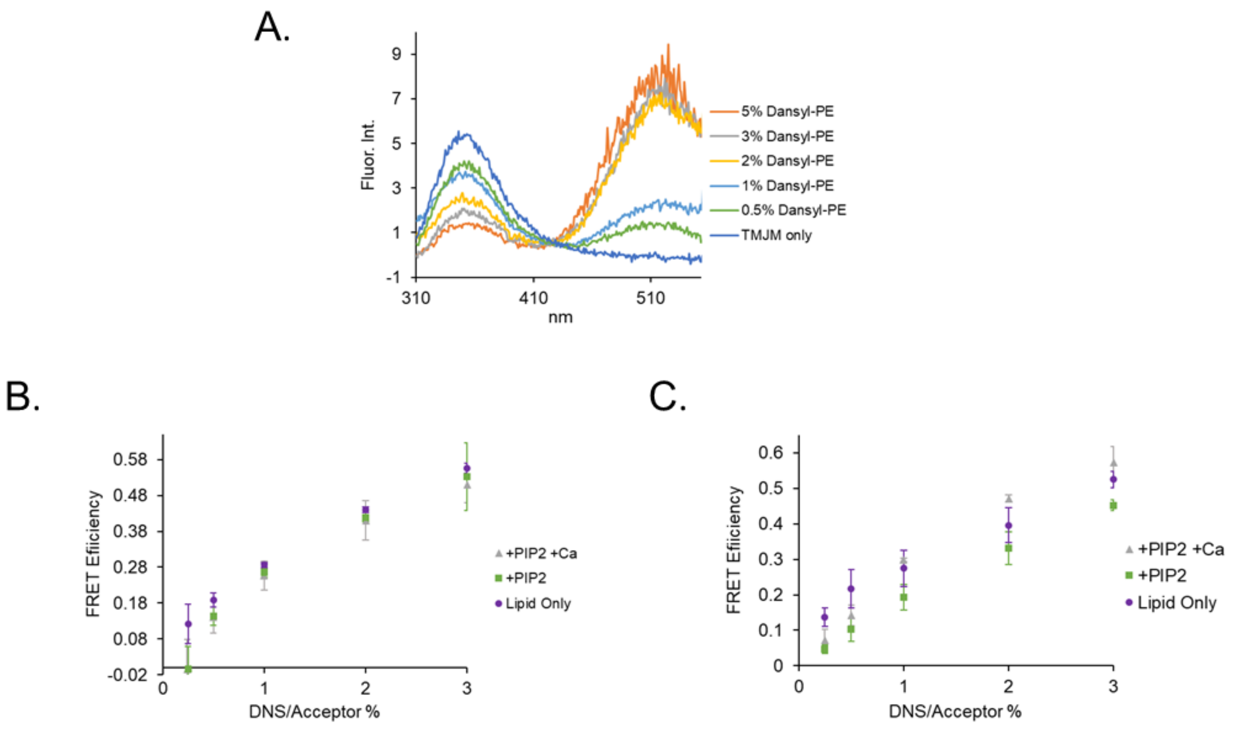

Supplemental Figure 7. A. Representative emission spectra of Trp and Dansyl showing saturating amounts of FRET at ~2% DNS-PE. **B.** FRET efficiencies of 1 µM TMJM in LUVs with 0-3% DNS-PE without PIP_2 or Ca^{2+} (purple), with 3% PIP_2 (green), and with 3% PIP_2 and 5 mM Ca^{2+} (grey) in 14:1 PC liposomes. **C.** FRET efficiencies of 1 µM TMJM in LUVs with 0-3% DNS-PE without PIP_2 or Ca^{2+} (purple), with 3% PIP_2 (green), and with 3% PIP_2 and 5 mM Ca^{2+} (grey) in 22:1 PC liposomes. Points are averages of 3 independent experiments ± S.D.

A.

B.

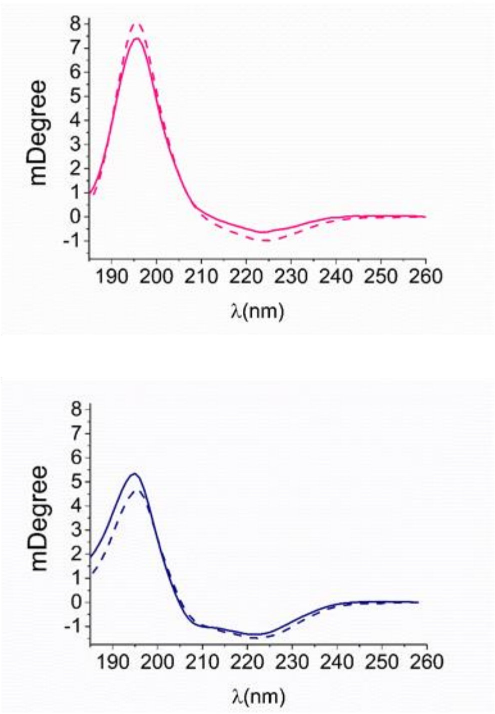

Supplemental Figure 8. Helical tilt in thin and thick bilayers is preserved upon addition of PIP_2. A. OCD spectra of TMJM in 22:1 PC with PIP_2 (dashed line) and without PIP_2 (solid line). Curves are averages of 3 independent experiments. **B.** OCD spectra of TMJM in 14:1 PC with PIP_2 (dashed line) and without PIP_2 (solid line). Curves are averages of 3 independent experiments.

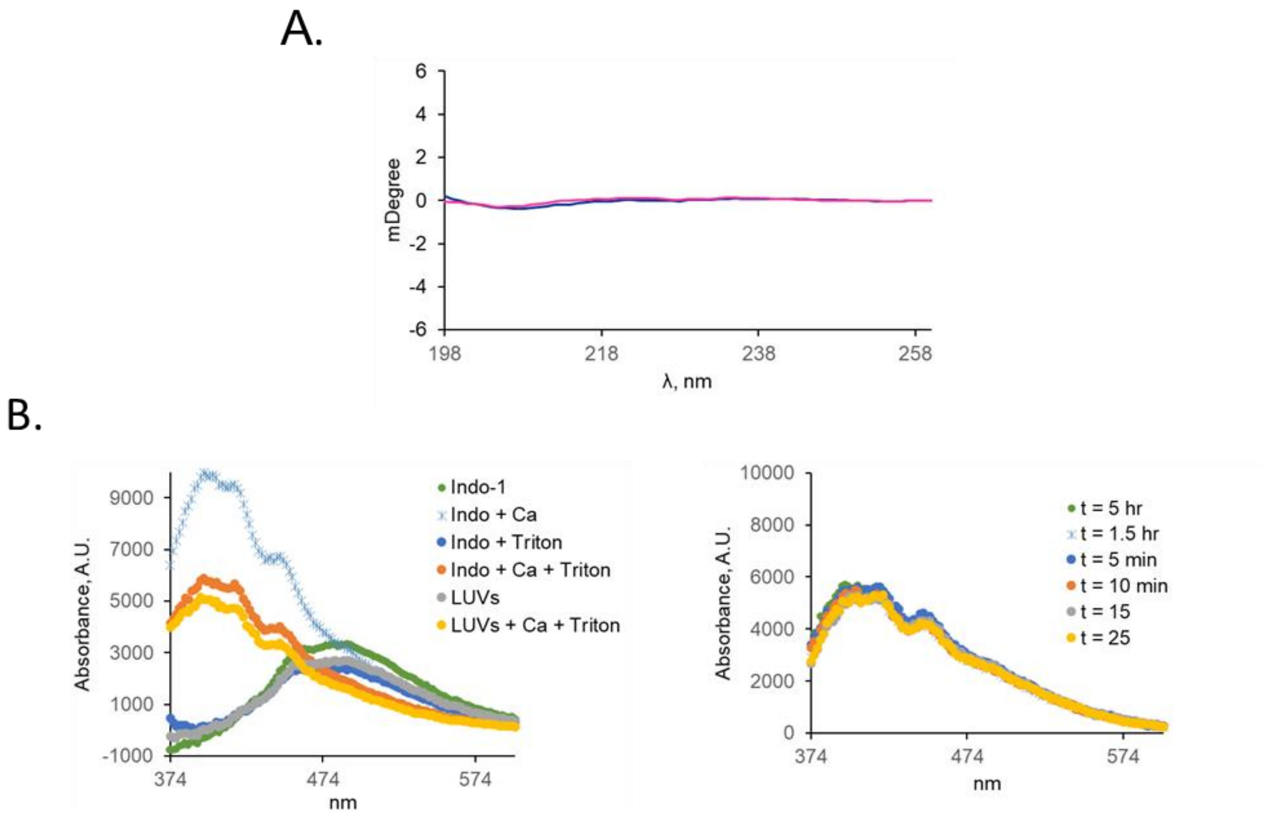

Supplemental Figure 9. A. Representative CD spectra of SDS-washed tubes indicating comparable levels of peptide were recovered for 22:1 PC (fuchsia) and 14:1 PC (navy) liposomes in fluorescence experiments. **B. (left)** Blue shift of encapsulated Indo-1 dye spectral maximum is observed after addition of 5 mM Ca^{2+}. **(right)** Calcium influx assays showing 5 mM Ca^{2+} crosses the membrane in saturating amounts within 25 minutes.

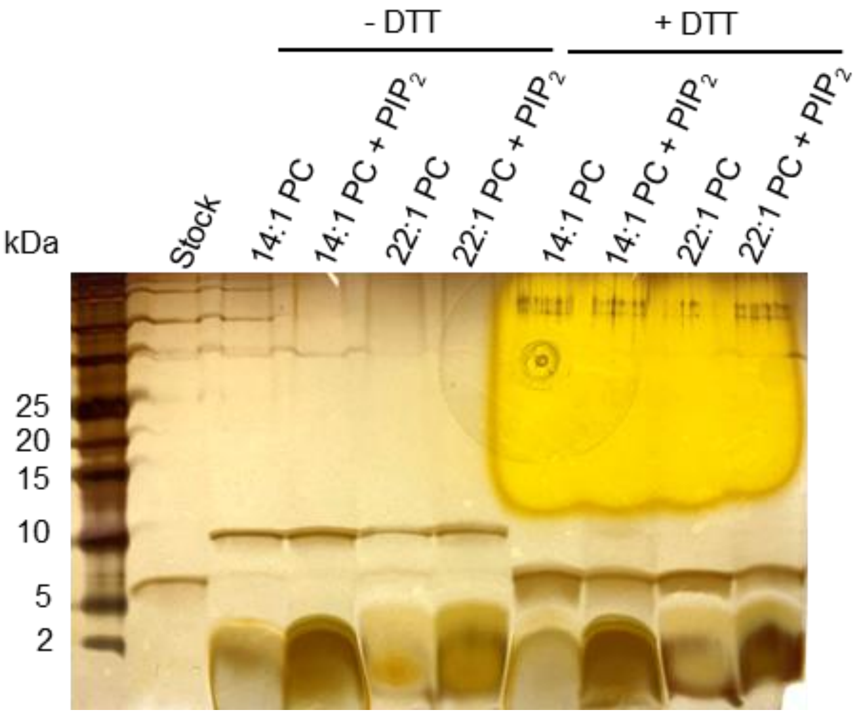

Supplemental Figure 10. Representative SDS PAGE gel of TMJM crosslinking as shown in Fig. 4C. Smears below 5 kDa are from lipids. Note: no smear is seen in first lane where no lipid was added. Yellow discoloration in upper-right portion of gel is from DTT, as the faint very high bands observed.
